# Nasal carriage of *Staphylococcus aureus* in children with grass pollen‐induced allergic rhinitis and the effect of polyvalent mechanical bacterial lysate immunostimulation on carriage status: A randomized controlled trial

**DOI:** 10.1002/iid3.584

**Published:** 2021-12-29

**Authors:** Kamil Janeczek, Andrzej Emeryk, Łukasz Zimmer, Ewa Poleszak, Michał Ordak

**Affiliations:** ^1^ Department of Pulmonary Diseases and Children Rheumatology Medical University of Lublin Lublin Poland; ^2^ Department of Applied and Social Pharmacy Medical University of Lublin Lublin Poland; ^3^ Department of Pharmacodynamics Centre for Preclinical Research and Technology, Medical University of Warsaw Warsaw Poland

**Keywords:** allergic rhinitis, bacterial lysate, children, nasal colonization, *Staphylococcus aureus*

## Abstract

**Background:**

Numerous studies indicate that *Staphylococcus aureus* (*S. aureus*) colonizing the nasal cavity plays a role in the pathogenesis of allergic rhinitis (AR). This bacterium is able to produce a variety of toxins with superantigenic properties that can exacerbate allergic inflammation.

**Objective:**

The objective of the study was to evaluate the ability of polyvalent mechanical bacterial lysate (PMBL) to eliminate *S. aureus* nasal carriage in children with grass pollen‐induced AR.

**Methods:**

This randomized, double‐blind, placebo‐controlled study included 80 children aged 5–17 years with seasonal AR (SAR). At the randomization visit and after 12 weeks of the study, a swab was taken from the region of the middle nasal meatus. Standard microbiology culture and identification techniques were used to analyze the swab contents.

**Results:**

Nasal colonization by *S. aureus* was confirmed in 29 children (42%), with *Moraxella catarrhalis* in three participants (4.4%). Physiological flora was detected in 37 children. No statistically significant differences were observed between the two measurement points in both the PMBL and placebo groups with respect to the number of patients whose nasal swab cultures showed a growth of *S. aureus* (*p* = 1). Both groups also showed no significant changes in the mean number of *S. aureus* colonies in nasal swab cultures taken at baseline and after 12 weeks of the study (PMBL group *p* = .41; placebo group *p* = .16).

**Conclusion:**

Almost every second child with SAR is *S. aureus* nasal carrier. Sublingual administration of PMBL in children with grass pollen‐induced AR did not affect *S. aureus* nasal colonization. Therefore, PMBL should not be used for the eradication of *S. aureus* from the nasal cavity.

## Graphical abstract

Patients with allergic rhinitis are more frequently colonized with nasal Staphylococcus aureus than healthy subjects, and those with positive Staphylococcus aureus results have more severe allergic rhinitis. The reduction of symptoms of seasonal allergic rhinitis in children resulting from polyvalent mechanical bacterial lysate therapy is not due to the eradication of Staphylococcus aureus from the nose.

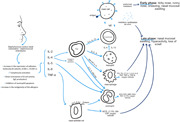

## 1 INTRODUCTION

Within the last few decades, there has been a steady increase in the prevalence of allergic diseases. The most common of these in the pediatric population is allergic rhinitis (AR), which affects approximately 40% of children. The disease is primarily associated with symptoms such as nasal congestion, rhinorrhea, nasal itching, and sneezing.^1^, ^2^, ^3^ However, AR also involves an impairment of the patient's daily functioning in home and school life and a risk of other diseases such as asthma, rhinosinusitis, and middle ear infections.^4^, ^5^, ^6^, ^7^

The main factor modifying the course of AR is exposure to sensitizing allergens. In children, nasal symptoms are most often induced by house dust mites, followed by grass, tree, and weed pollen. Nonallergenic factors such as air pollution, odors, exercise, and temperature fluctuations may also affect the symptoms.^8^ Furthermore, it has been shown that nasal carriage of *Staphylococcus aureus* (*S. aureus*) may promote local inflammation and thus exacerbate AR symptoms.^9^, ^10^, ^11^, ^12^ Eradication of the carriage of this bacterium was associated with decreased symptom severity of AR.^12^ Staphylococcal colonization may also influence the course of other allergic diseases such as asthma and atopic dermatitis. Nasal *S. aureus* or specific IgE in serum against *S. aureus* enterotoxins were associated with increased wheeze frequency, increased risk of asthma prevalence, greater severity of symptoms, and more frequent exacerbations.^13^, ^14^ Higher SCORAD scoring was recorded in children with atopic dermatitis and nasal *S. aureus* colonization compared with children with no nasal colonization.^15^ Thus, *S. aureus* colonizing nasal mucosa has an influence on the development and severity of allergic diseases.

Polyvalent bacterial lysates (PBLs) are oral, sublingual, intranasal, or injectable immunostimulating nonspecific vaccines, which are composed of combinations of extracts from various bacteria, most commonly being the etiological factors responsible for acute and chronic respiratory tract infections.^16^ Depending on the extraction method we divide them into chemical (PCBL) and mechanical (PMBL) lysates. PCBLs consist of antigenic molecules structurally damaged by protein denaturation in an alkaline environment. PMBLs, on the other hand, are characterized by less damage to bacterial antigens and less chemical contamination. Therefore, PMBLs may exert a greater clinical effect than PCBLs.^17^, ^18^ PBLs are capable of activating innate and adaptive immune response. They stimulate dendritic cells, T and B cells, IgA secretion, as well as the synthesis of opsonizing antibodies directed against administered bacterial antigens.^19^ PBLs prevent recurrent respiratory tract infections. Moreover, they reduce their severity, duration, and indications for antibiotics.^20^, ^21^ Recent studies have also highlighted their benefits in patients with allergic diseases. PBLs have been shown to be effective in the prevention and treatment of atopic dermatitis in children,^22^, ^23^ improve the clinical course of AR,^24^, ^25^, ^26^, ^27^ and reduce asthma exacerbations.^28^

In view of the benefits of PBLs in allergic diseases and their effect on improving the efficacy of the mucosa‐related immune system in eliminating pathogens, a hypothesis was made that the improvement in the clinical course of AR in children treated with PMBLs might be due to a reduction in the nasal carriage of *S. aureus*.

The objective of this study was to assess the frequency of nasal *S. aureus* carriage and the results of PMBL administration for eliminating bacterial nasal colonization by the *S. aureus* in children with grass pollen‐induced AR.

## 2 METHODS

### 2.1 Study design

This randomized, double‐blind, placebo‐controlled study was conducted according to the Declaration of Helsinki principles. The study protocol and informed consent form were approved by the Bioethics Committee of the Medical University of Lublin (resolution number KE‐0254/41/2018 of 22 February 2018). Pharmaceutical companies had no involvement in this project.

The first part of the study was conducted in three clinical centers in Poland between April and August 2018. The main objective of this study from 2018 was to assess the efficacy of PMBL therapy in improving the clinical course of seasonal AR (SAR) caused by grass pollen allergens in children during the grass pollen season. The results of this study confirming the effect of PMBL in reducing the severity of SAR symptoms in children have been published previously in JACI: in Practice.^27^ In the study, nasal swabs were taken for bacterial culture in a subgroup of 38 patients, with suspicion that any reduction in the severity of SAR symptoms might be due to eradication of nasal *S. aureus* carriage. To verify this hypothesis, the study was repeated in 2020, including the missing number of patients according to the sample size calculation (see below).

Thus, the primary objective of this study was to examine the efficacy of three‐month PMBL therapy in reducing *S. aureus* nasal carriage in children with SAR caused by grass pollen allergens during the grass pollen season. The secondary objectives were to assess the frequency of *S. aureus* nasal carriage among children with SAR, the consumption of oral H1‐antihistamines and intranasal corticosteroids, and to assess the safety profile of the applied intervention.

### 2.2 Patients

Eligible participants were children aged 5–17 years with grass pollen‐induced AR recognized and treated according to current ARIA (Allergic Rhinitis and its Impact on Asthma) recommendations.^29^

All the inclusion and exclusion criteria were published recently.^27^ Children were recruited for the study in late April 2018 and 2020, that is, before the start of the grass pollen season in Poland (Children's University Hospital in Lublin, Allergy Clinic). All patients and their parents gave written informed consent.

### 2.3 Interventions

The characteristics of the PMBL used in the study, the description of the preparation of the placebo, and the sublingual tablet administration schedule have been previously published.^27^

### 2.4 Randomization and masking

We described the randomization process in the previous article.^27^

### 2.5 Study protocol

**Figure 1:**
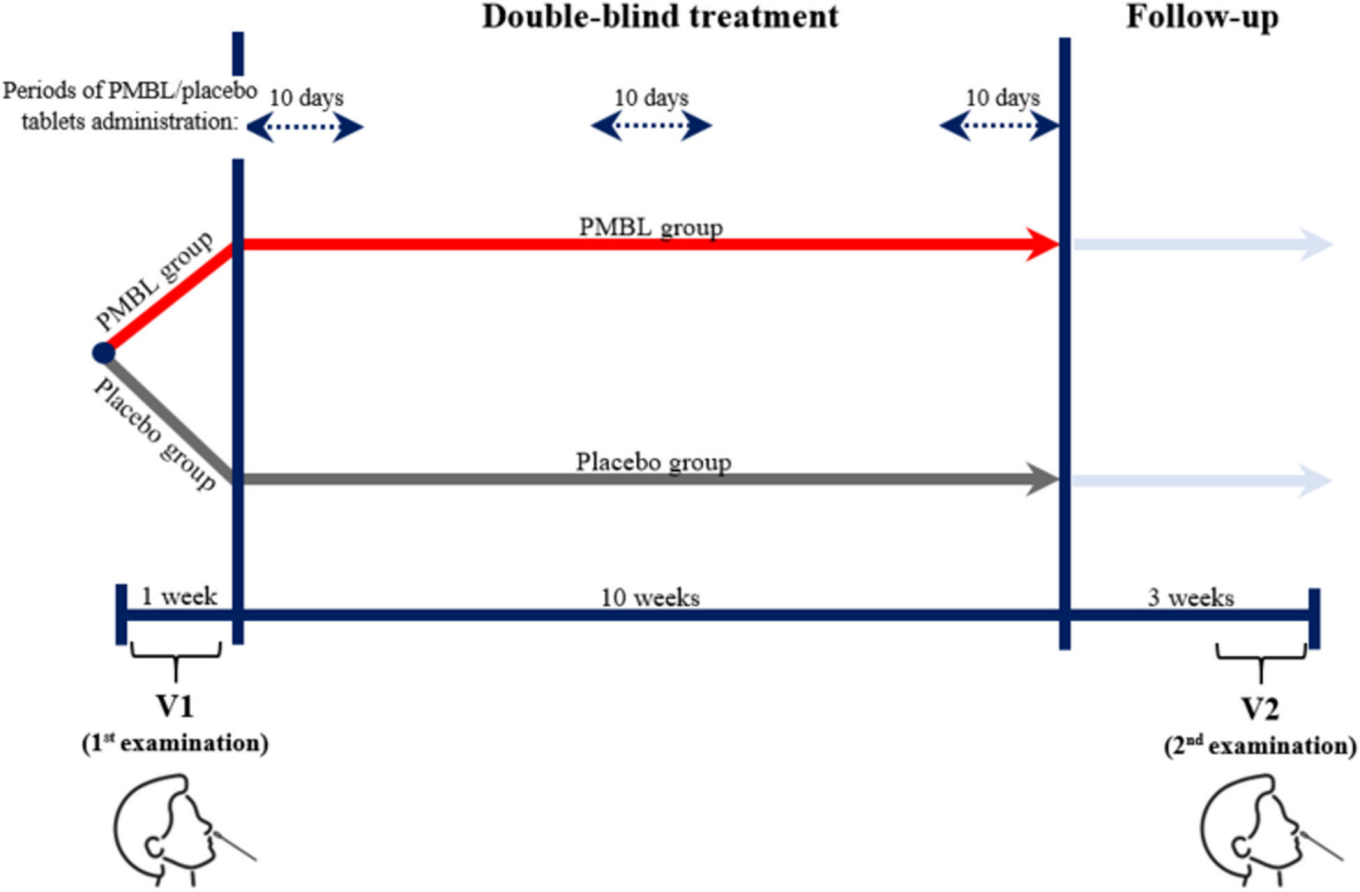
Study design

The study consisted of two visits, the first before the beginning of the grass pollen season (screening/randomization visit, first examination) and the second after 12 weeks of the study (3 weeks before the end of the grass pollen season, second examination) (Figure 1). The 95% method was used to determine the time frame of the grass pollen season, based on retrospective measurements of the concentration of grass pollen grains in the ambient air for south‐eastern Poland.^30^

Beginning May 1, 2018 or May 1, 2020, parents administered a sublingual PMBL or placebo tablet to their children and recorded additional medications taken in the patient diary. Patients had the possibility to take oral H1‐antihistamine (desloratadine) and intranasal corticosteroid (mometasone furoate) on demand to relieve SAR symptoms. Desloratadine was the drug of the first choice, and if there was no improvement the patient could additionally take intranasal corticosteroid for 10–14 days.^29^

### 2.6 Nasal smear collection procedure and evaluation of nasal bacterial flora

At the randomization visit (first examination) and after 12 weeks of the study (second examination) a swab was taken from the region of the middle nasal meatus using sterile cotton‐tipped swabs (Deltalab). The collected material was placed in a tube and transferred within 30 min to the laboratory of the University Children's Hospital in Lublin (DIAGNOSTYKA Sp. z o.o.) were processed on the same day. Standard microbiology culture and identification techniques were used to analyze the swab contents.^31^, ^32^ After the incubation period, the microbiologist counted the bacterial colonies and described the bacterial growth as: single (1–10 colonies), sparse (11‐20 colonies), medium (21–30 colonies), numerous (>30 colonies), or abundant (uncountable numbers of colonies).^33^, ^34^

### 2.7 Sample size

The sample size was determined based on previous studies on the effects of fusidic acid and topical nasal mupirocin on nasal *S. aureus* eradication in patients with AR.^12^, ^35^ It was estimated that 15 patients with *S. aureus* growth in nasal swab cultures should be included in both groups. Assuming that nasal *S. aureus* carriage occurs in approximately 40% of patients with AR, approximately 76 children should have been included in the study.^9^, ^35^

### 2.8 Statistical analysis

The IBM SPSS Statistics 25 package was used to perform the statistical analysis. McNemar's test was used to assess the presence of statistically significant differences in the presence of *S. aureus* in nasal swab cultures between the two measurement points. The Wilcoxon test, on the other hand, was used to check whether there were statistically significant differences in the intensity of *S. aureus* growth in nasal swab cultures between the two visits. The *χ* ^2^ test was used to determine if there is a significant relationship between the nominal variables. *p* value <.05 was considered statistically significant.

The intent‐to‐treat (ITT) population, defined as all patients who were randomized, received at least one tablet of a study drug, and had at least one post‐baseline assessment, was used for analyses.

## 3 RESULTS

### 3.1 Participant flow

**Figure 2:**
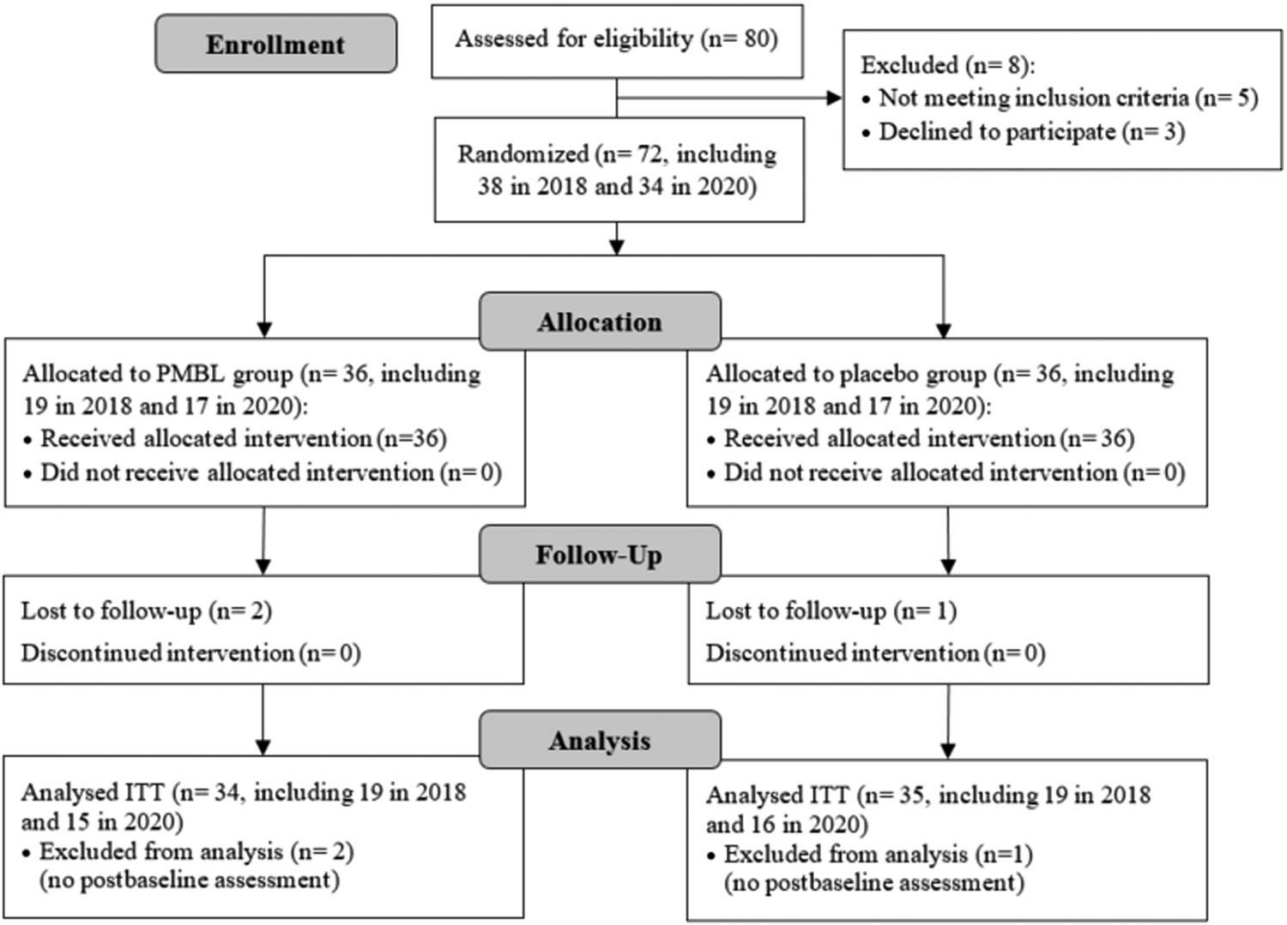
Participant flow diagram. PMBL, polyvalent mechanical bacterial lysate

Eighty children were enrolled in the study, including 38 patients who participated in a 2018 study evaluating the efficacy of PMBL therapy in reducing the severity of SAR symptoms and from whom nasal swabs were then collected for culture.^27^ Figure 2 shows the flow of participants through the trial (Figure 2). There were no statistically significant differences between the compared groups in terms of age, sex, place of residence, and allergies (Table 1).

**Table 1:** Demographic characteristics of participants

	PMBL group (*n* = 36)	Placebo group (*n* = 36)	*p* value
**Sex, *n* (%)**			
Male	21 (58.3)	19 (52.8)	.81
Female	15 (41.7)	17 (47.2)	
**Age (y)** a	9.42 (3.07)	9.25 (3.06)	.82
**Place of residence, *n* (%)**			
Village	17 (47.2)	18 (50)	1
City	19 (52.8)	18 (50)	
**Sensitizing allergen, *n* (%)**			
Grasses	36 (100)	36 (100)	‐
Cereals	31 (86.1)	31 (86.1)	1
Trees	18 (50)	16 (44.4)	.81
Weeds	8 (22.2)	6 (16.7)	.77
House dust mite	25 (69.4)	22 (61.1)	.62
Pet dander	10 (27.8)	7 (19.4)	.58

Abbreviation: PMBL, polyvalent mechanical bacterial lysate.

^a^ Mean (*SD*)

None of the patients from whom nasal swabs were taken for culture required antibiotic therapy one month before and throughout the study.

### 3.2 Primary outcome

Nasal colonization by *S. aureus* was confirmed in 29 children (42%) (15 from the PMBL group and 14 from the placebo group), with *Moraxella catarrhalis* in three participants (4.4%) (two from the PMBL group and one from the placebo group). Physiological flora was detected in 37 children (53.6%) (17 from the PMBL group and 20 from the placebo group) (Table 2).

**Table 2:** Effectiveness of treatment with PMBL in reducing *Staphylococcus aureus* nasal carriage

Examination	PMBL group (*n* = 34)			Placebo group (n = 35)		
	*S. aureus n* (%)	Other bacteria *n* (%)	Physiological flora n (%)	*S. aureus n* (%)	Other bacteria *n* (%)	Physiological flora *n* (%)
**First**	15 (44.1)	2^a^ (5.9)	17 (50)	14 (40)	1^a^ (2.9)	20 (57.1)
**Second**	14 (41.2)	2^a^ (5.9)	18 (52.9)	15 (42.9)	1^a^ (2.9)	19 (54.2)
***p***	1	1	1	1	1	1

Abbreviation: PMBL, polyvalent mechanical bacterial lysate.

^a^ *Moraxella catarrhalis*.

No statistically significant differences were observed between the two measurement points in both the placebo and PMBL groups with respect to the number of patients whose nasal swab cultures showed a growth of *S. aureus* (*p* = 1) (Table 2). The groups compared were not shown to be statistically significantly different in terms of the evaluated variable at the first (*χ* ^2^(1) = 0.06, *p* = 1) and second (*χ* ^2^(1) = 0.02, *p* = 1) measurement point.

In both the placebo and PMBL groups, there were no statistically significant differences in the mean number of *S. aureus* colonies in nasal swab cultures collected at baseline and after 12 weeks of the study (*p* = .41, *p* = .16, respectively) (Table 3). The groups compared were not statistically significantly different in the mean number of *S. aureus* colonies in nasal swab cultures at the first (*χ* ^2^(2) = 0.5, *p* = .78) and second (*χ* ^2^(2) = 0.55, *p* = .76) measurement point.

**Table 3:** Effectiveness of treatment with PMBL in reducing bacterial colonies count of *Staphylococcus aureus* colonizing the nasal mucosa

PMBL group			
Number of bacterial colonies	First examination (*n* = 15)	Second examination (*n* = 14)	*p*
1–10	0	0	.16
11–20	3 (20)	2 (14.3)	
21–30	11(73.3)	10 (71.4)	
>30	1 (6.7)	2 (14.3)	
Uncountable	0	0	

**Placebo group**			
**Number of bacterial colonies**	**First examination** (*n* = 14)	**Second examination** (*n* = 15)	***p***
1–10	0	0	.41
11–20	3 (21.4)	3 (20)	
21–30	9 (64.3)	11 (73.3)	
>30	2 (14.3)	1 (6.7)	
Uncountable	0	0	

Abbreviation: PMBL, polyvalent mechanical bacterial lysate.

### 3.3 Secondary outcome

The consumption of oral H1‐antihistamines and intranasal corticosteroids was lower in the PMBL group compared to the placebo group by 29% and 33%, respectively. A comparable safety profile of PMBL and placebo has been demonstrated.

## 4 DISCUSSION

This study was designed to assess the frequency of nasal *S. aureus* carriage and the results of PMBL administration for eliminating bacterial nasal colonization by the *S. aureus* in children with grass pollen‐induced AR. Our study represents the first clinical effort to evaluate the applicability of PMBL to the eradication of nasal carriage of *S. aureus* in children with SAR.

The main bacterial flora of nasal cavity includes coagulase‐negative staphylococci (10%–80%), aerobic diphtheroids (5%–70%), and *S. aureus* (5%–35%).^36^ There are factors that modify the composition of this flora, such as diabetes mellitus, dialysis, and smoking.^37^, ^38^ The anterior nares of the nose are the most common location of *S. aureus* in our body. We can distinguish three *S. aureus* nasal carriage patterns in healthy subjects: persistent, intermittent, or non‐*S. aureus* nasal carriers. Wertheim et al.^39^ estimated that approximately 20% of healthy subjects are persistent *S. aureus* nasal carriers, 30% are intermittent and 50% are non‐carriers. Persistent carriage is more commonly found in children than in adults.^40^

The *S. aureus* nasal carriage rate among patients with AR compared with healthy individuals was addressed in previous studies. There is some controversy regarding this topic. However, most studies indicate that the prevalence of the *S. aureus* nasal carriage in patients with AR is higher than that seen in the healthy population.^9^, ^10^, ^11^, ^32^, ^35^, ^41^, ^42^, ^43^ Carriage of this bacterium is found in up to 40% of patients with respiratory allergies.^9^, ^35^ Our study seems to support these data, showing that the prevalence of *S. aureus* nasal carriage in children with SAR is 41%. Such frequent colonization of the nasal cavity by *S. aureus* in patients with AR may be due to: frequent hand‐to‐nose contact caused by nose‐picking or blowing, frequent antibiotic therapy, use of staphylococcal‐contaminated nasal sprays, high glucose content in nasal secretions, impaired mucociliary clearance, damage to nasal mucosa which increases bacterial adhesion and less host defense.^9^, ^35^, ^44^, ^45^

**Figure 3:**
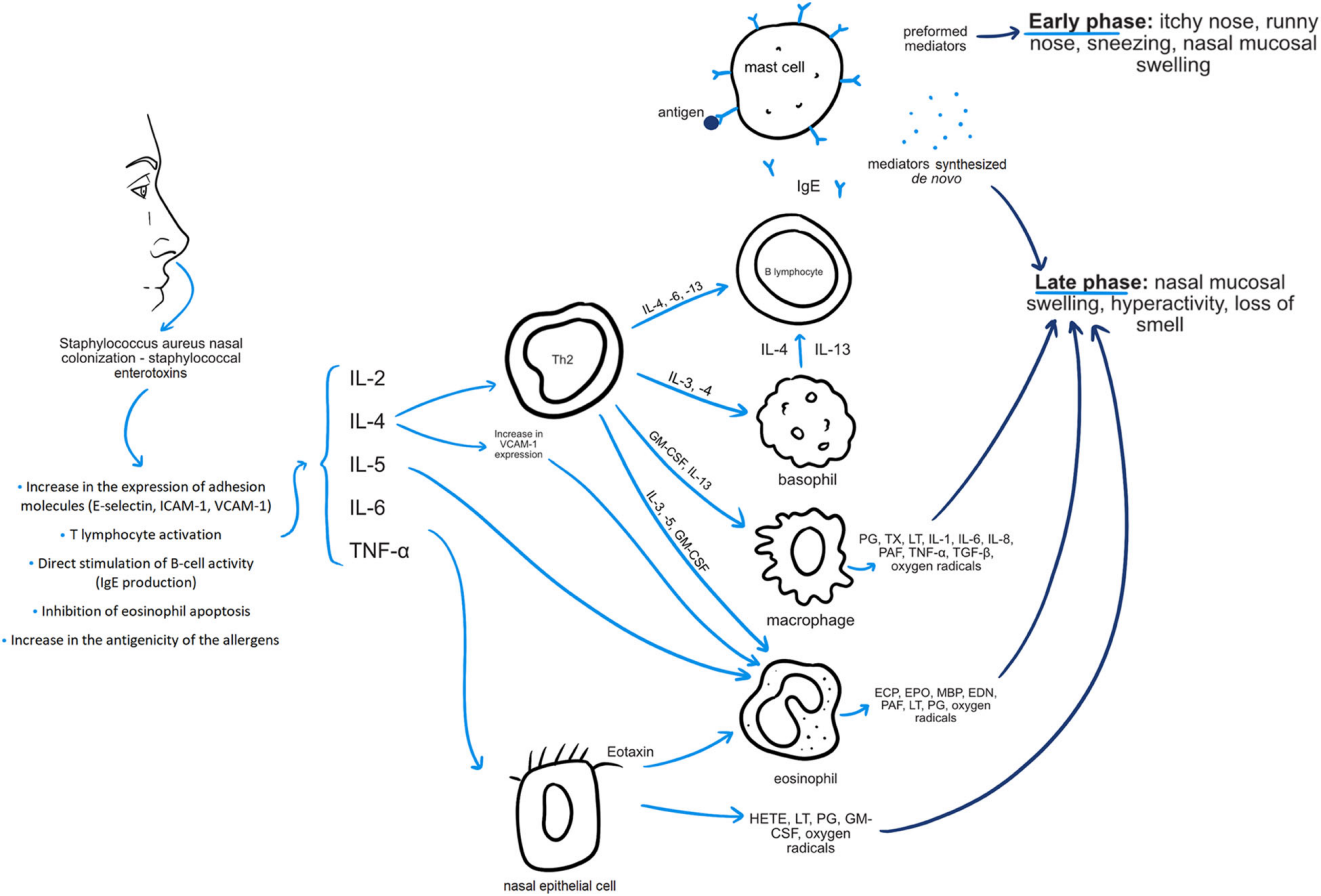
Mechanisms of nasal *Staphylococcus aureus* and its toxins on allergic rhinitis—own elaboration based on [9–11,44,46,49]. ECP, eosinophil cationic protein; EDN, eosinophil‐derived neurotoxin; EPO, eosinophil peroxidase; GM‐CSF, granulocyte‐macrophage colony‐stimulating factor; HETE, hydroxyeicosatetraenoic acid; ICAM‐1, intercellular adhesion molecule 1; IL, interleukin; LT, leukotriene; MBP, major basic protein; PAF, platelet‐activating factor; PG, prostaglandin; TGF‐β, transforming growth factor‐beta; Th2, T‐helper type‐2; TNF‐α, tumor necrosis factor‐alpha; TX, thromboxane; VCAM‐1, vascular cell adhesion molecule 1

It has been suggested that *S. aureus* colonizing the nasal cavity may play a role in the pathogenesis of AR. This bacterium is able to produce a variety of toxins with superantigenic properties, which may influence the activity of immunomodulatory and pro‐inflammatory cells.^46^ The effect of this is the promotion of local inflammation and, consequently, exacerbated symptoms of allergic disease. Shimori et al.^9^ evaluated the frequency of nasal *S. aureus* carriage in patients with perennial AR (PAR) and its impact on the clinical course of the disease. The researchers showed that half of the allergic patients were colonized by *S. aureus* and nasal symptom scores were significantly higher in these patients compared with the *S. aureus*‐negative group. They also found that peripheral blood mononuclear cells from patients with PAR produced lower amounts of INF‐Ƴ and larger amounts of Th2‐type cytokines (IL‐4, IL‐5) after stimulation with staphylococcal exotoxins. It is worth mentioning here that such a constellation of changes in cytokine concentrations plays an important role in the pathogenesis of allergic diseases. IL‐4 contributes to the production of asIgE by B lymphocytes, whilst IL‐5 contributes to nasal infiltration by eosinophils.^47^, ^48^ Similar results were obtained by Refaat et al.^11^ showing a positive correlation between nasal *S. aureus* counts and severity of sneezing, as well as immunological parameters (serum total IgE, serum asIgE, nasal total IgE, and nasal IL‐4) in patients with PAR. The German researchers observed greater nasal obstruction, hypersecretion, and irritation in allergic *S. aureus* carriers when compared with allergic non‐carriers, but this difference did not reach statistical significance.^10^ This group had significantly higher levels of IL‐13, eosinophil cationic protein, total IgE, and lower levels of IFN‐Ƴ in nasal lavage fluid. There were no significant differences in serum total IgE between the compared groups. Therefore, the researchers concluded that *S. aureus* is responsible for the local stimulation of IgE production. The effect of nasal exposure to staphylococcal toxins was also evaluated in a murine model of AR.^49^ This exposure was associated with increased levels of total IgE, asIgE, IL‐4, IL‐5 in blood and nasal eosinophilia as determined by histological examination. Similar observations apply to patients with other allergic diseases.^50^, ^51^ Figure 3 provides a simplified diagram showing the involvement of *S. aureus* colonizing the nasal cavity in the pathomechanism of AR (Figure 3).

In the January 2021 issue of JACI: in Practice, we published the results of a study demonstrating that sublingually administered PMBL improves the clinical course of SAR in children sensitized to grass pollen allergens.^27^ Based on the data obtained, we concluded that PMBL reduces the allergic response of Th2 cells. Concurrently, we point out that the mechanism of action of PMBL in SAR is probably more complex. Searching for other mechanisms in which PMBL improves the clinical course of SAR, while considering the influence of bacterial lysates in improving the effectiveness of the mucosa‐related immune system in eliminating pathogens and the involvement of *S. aureus* in the pathogenesis of allergic diseases, we decided to conduct a study to evaluate the ability of PMBL to eradicate nasal *S. aureus*.

In the present study, it was established that PMBL immunostimulation in children with SAR did not affect nasal *S. aureus* colonization. This therapy has not been shown to affect eradication of *S. aureus* from the nasal cavity or to reduce the mean number of *S. aureus* colonies in nasal swab cultures. Thus, the beneficial effect of PMBL on the clinical course of SAR in children confirmed in an earlier study^27^ is not due to the ability of this drug to eradicate nasal *S. aureus* carriage.

Only one study is available evaluating the use of bacterial lysate in eliminating bacterial nasal colonization.^52^ Zagolski et al.^52^ enrolled adults with confirmed nasal or pharyngeal bacterial colonization by *Streptococcus pneumoniae*, *Haemophilus influenzae*, *S. aureus*, or β‐hemolytic streptococci. Patients took PCBL (Luivac®) or oral personalized autovaccine for 2 months. Reassessment of nasal swabs after 16 weeks showed that PCBL reduced *Haemophilus influenzae* and *Streptococcus pneumoniae*, while autovaccine reduced Streptococcus pneumoniae and β‐hemolytic streptococci. *S. aureus* colonization did not respond to either treatment method, which in terms of bacterial lysates is in line with the results of our study. Two studies have evaluated the effect of intranasal corticosteroids used in AR therapy on carriage of *S. aureus*.^53^, ^54^ In the first (and one of the few), the frequency of *S. aureus* nasal carriage was comparable between patients with and without AR (21.4% vs. 15.9%).^53^ The researchers found no relationship between carrying this bacterium and AR symptoms, but the severity of AR symptoms was not assessed. The *S. aureus* nasal carriage rate has decreased after treatment with intranasal fluticasone propionate but this decrease was not statistically significant. Similarly, no significant reduction in *S. aureus* carriage rate was observed in the second study under monthly mometasone furoate nasal spray therapy in patients with PAR.^54^ On the contrary, Hessam and Elazab^12^ demonstrated that nasal symptoms of AR increase with nasal colonization with *S. aureus* and improve after its eradication with topical fusidic acid.

*S. aureus* has been found to be hard to eradicate from the nasal cavity, particularly in patients with AR. In their work, Zagolski et al.^52^ ponder why PCBL is effective in some patients with the same bacteria colonizing the same anatomical region and others it is not, at the same time citing as one of the possible reasons high phenotypic diversity in *S. aureus* strains residing in the upper respiratory tract. Other possible causes in patients with AR include decreased immune adhesive function of leukocytes or decreased activity of regulatory T cells.^55^

The work has some limitations. The first possible limitation is the lack of detection of *S. aureus* genetic material in patient samples. A less sensitive method such as bacteriological culture was used to detect the carrier status. However, the culture method detects live microorganisms and not residual genetic material of bacteria after infection. Another potential limitation of this study is the evaluation of only nasal carriage of *S. aureus*, since this bacterium can also colonize other areas of our body, such as the throat or skin. Further studies are required to determine what role the eradication of nasal *S. aureus* carriage may have in preventing the aggravation of AR.

## 5 CONCLUSION

Almost every second child with SAR is *S. aureus* nasal carrier. Sublingual administration of PMBL in children with grass pollen‐induced AR did not affect *S. aureus* nasal colonization. Therefore, PMBL should not be used for the eradication of *S. aureus* from the nasal cavity.

## AUTHOR CONTRIBUTIONS

Kamil Janeczek: conceptualization, data curation, formal analysis, investigation, methodology, supervision, writing original draft. Andrzej Emeryk: conceptualization, data curation, investigation, methodology, writing original draft. Łukasz Zimmermmer: conceptualization, data curation, methodology, writing original draft. Ewa Poleszak: conceptualization, data curation, methodology, writing original draft Michał Ordak: conceptualization, data curation, formal analysis, methodology, writing original draft.

## Data Availability

The datasets used and/or analyzed during the current study are available from the corresponding author on reasonable request.
